# Cytotoxicity Study of UV-Laser-Irradiated PLLA Surfaces Subjected to Bio-Ceramisation: A New Way towards Implant Surface Modification

**DOI:** 10.3390/ijms22168436

**Published:** 2021-08-05

**Authors:** Konrad Szustakiewicz, Bartłomiej Kryszak, Paulina Dzienny, Błażej Poźniak, Marta Tikhomirov, Viktoria Hoppe, Patrycja Szymczyk-Ziółkowska, Włodzimierz Tylus, Michał Grzymajło, Agnieszka Gadomska-Gajadhur, Arkadiusz J. Antończak

**Affiliations:** 1 Department of Polymer Engineering and Technology, Faculty of Chemistry, Wrocław University of Science and Technology (WUST), Wyb. Wyspiańskiego 27, 50-370 Wrocław, Poland; michal.grzymajlo@pwr.edu.pl; 2 Laser and Fiber Electronics Group, Faculty of Electrical Engineering, Wrocław University of Science and Technology, 50-370 Wrocław, Poland; paulina.dzienny@pwr.edu.pl (P.D.); arkadiusz.antonczak@pwr.edu.pl (A.J.A.); 3 Department of Pharmacology and Toxicology, Faculty of Veterinary Medicine, Wrocław University of Environmental and Life Sciences, ul. Norwida 25, 50-375 Wrocław, Poland; blazej.pozniak@upwr.edu.pl (B.P.); marta.tikhomirov@upwr.edu.pl (M.T.); 4 Centre for Advanced Manufacturing Technologies, Faculty of Mechanical Engineering, Wrocław University of Science and Technology (WUST), Łukasiewicza 5, 50-370 Wrocław, Poland; viktoria.hoppe@pwr.edu.pl (V.H.); patrycja.e.szymczyk@pwr.edu.pl (P.S.-Z.); 5 Department of Advanced Material Technologies, Faculty of Chemistry, Wrocław University of Science and Technology, 50-370 Wrocław, Poland; wlodzimierz.tylus@pwr.edu.pl; 6 Faculty of Chemistry, Warsaw University of Technology, ul. Noakowskiego 3, 00-664 Warsaw, Poland; agadomska@ch.pw.edu.pl

**Keywords:** polymers, poly(L-Lactide), laser modification, extrusion casting, selective biomineralization, fibroblasts, macrophage-like cells, cell response

## Abstract

In this research we subjected samples of poly(L-lactide) (PLLA) extruded film to ultraviolet (193 nm ArF excimer laser) radiation below the ablation threshold. The modified film was immersed in Simulated Body Fluid (SBF) at 37 °C for 1 day or 7 days to obtain a layer of apatite ceramic (CaP) coating on the modified PLLA surface. The samples were characterized by means of optical profilometry, which indicated an increase in average roughness (Ra) from 25 nm for the unmodified PLLA to over 580 nm for irradiated PLLA incubated in SBF for 1 day. At the same time, the water contact angle decreased from 78° for neat PLLA to 35° for irradiated PLLA incubated in SBF, which suggests its higher hydrophilicity. The obtained materials were investigated by means of cell response fibroblasts (3T3) and macrophage-like cells (RAW 264.7). Properties of the obtained composites were compared to the unmodified PLLA film as well as to the UV-laser irradiated PLLA. The activation of the PLLA surface by laser irradiation led to a distinct increase in cytotoxicity, while the treatment with SBF and the deposition of apatite ceramic had only a limited preventive effect on this harmful impact and depended on the cell type. Fibroblasts were found to have good tolerance for the irradiated and ceramic-covered PLLA, but macrophages seem to interact with the substrate leading to the release of cytotoxic products.

## 1. Introduction

Because of its wide range of applications, especially in regenerative medicine, poly(L-lactide) (PLLA) is one of the most popular bioresorbable polymers. Scientists from around the world find it very attractive to use for the preparation of vascular stents [1], and it also opens up various new possibilities in bone tissue engineering [2]. All this is possible thanks to its good biodegradability and biocompatibility [3]. However, the polymer also has some disadvantages, e.g., its insufficient mechanical properties, slow crystallization rate [4], and also its high hydrophobicity and lack of bioactivity. In addition, during its degradation, PLLA releases products that may cause an inflammatory response in adjacent tissues [5].

In order to overcome the problems related to the application of this polymer in regenerative medicine, several strategies for the modification of PLLA have been undertaken. The chosen strategy mainly depends on the potential application. For bone tissue engineering, the polymer is usually doped with apatite ceramics (the most common being hydroxyapatite or tricalcium phosphate) which support appropriate osteoconductivity, biocompatibility, good bone-binding ability and bone regeneration [6]. This can be obtained thanks to the chemical and structural similarity between the mineral phase of native bone and the hydroxyapatite used as the PLLA dopant [7]. The PLLA/apatite materials can be obtained using the technique of extrusion [8,9,10], but also using solvent techniques including thermally induced phase separation [11,12,13,14], electrospinning [15,16] and solvent casting [17]. The addition of apatite ceramics into PLLA causes a decrease in water contact angle [14,18], which suggests that the material becomes more hydrophilic. Moreover, cell culture experiments for the apatite-doped PLLA show enhanced cell attachment and proliferation as well as alkaline phosphatase activity [15,19,20,21,22].

Another group of methods for combining PLLA with apatite ceramics is based on so called biomineralization. This process is usually referred to when inducing inorganic ions to crystallize and grow in vitro using organic templates [23,24]. In the case of the biomineralization methods, the PLLA surface is covered by calcium phosphates after immersion in Simulated Body Fluid (SBF) [25]. SBF, whose composition is almost equivalent to that of blood plasma, was first proposed by Kokubo to enhance bone-binding ability of implants [25]. The process of biomineralization, however, is very slow and might take up to a few weeks [26]. For this reason, the surface of the PLLA is usually activated by etching for the hydrolysis of the –COOH groups, which results in a negatively charged surface. After immersing in SBF, the negatively charged surface facilitates the accumulation of calcium cations and phosphate ions to form hydroxyapatite [2,27,28,29]. By adding the etching process before putting PLLA into SBF, one can reduce the time needed for a HA coating to develop [27].

There are several other methods for polymer biomineralization, such as electrodeposition [23,30], the lately described polydopamine modification of the PLLA fibers that facilitates mineralization in SBF [31], or the mineralization of the surface of PLLA induced by a UV laser [24]. This last method has been developed in our group. It opens up new possibilities due to the high selectivity of the process.

In the present research we show the cell response for biomineralized PLLA samples activated using a UV excimer laser (ArF, 193 nm). We prepared five types of samples: extruded reference PLLA film, UV-irradiated PLLA incubated in air for 1 day (24 h) and 7 days (168 h) and UV-irradiated PLLA incubated in SBF for 1 day and 7 days. We characterized the surface of the material by means of roughness, wettability and cell response.

## 2. Results and Discussion

### 2.1. Physicochemical Analysis/Topography of PLLA Films

**Figure 1:**
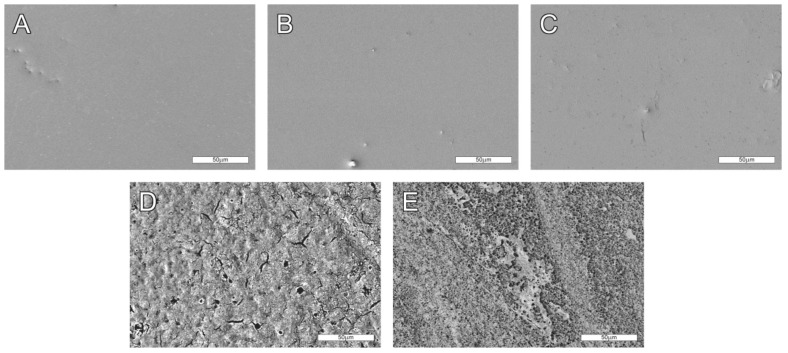
SEM images of analyzed samples. (**A**) Unmodified PLLA, (**B**,**C**) PLLA_UV_1d and PLLA_UV_7d, respectively, (**D**,**E**) PLLA_UV_SBF_1d and PLLA_UV_SBF_7d respectively. 500× magnification. Comment: The surface of unmodified PLLA (**A**) is smooth and does not change much after laser exposure after a day (**B**) and a week (**C**) of air incubation. However, after irradiation and incubation in SBF for one day (**D**) and one week (**E**), the surface is more markedly rough due to deposition of the apatite ceramic on it and hydrolytic degradation of the polymer.

**Figure 2:**
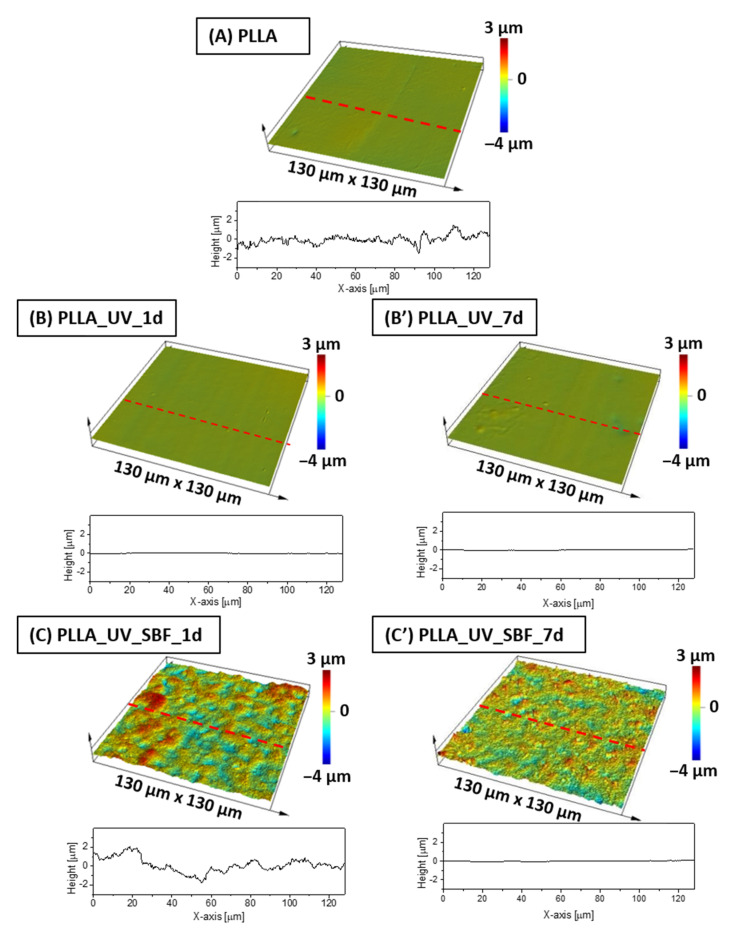
Surface profiles for (**A**) PLLA reference sample, (**B**,**B′**) PLLA after laser activation, (**C**,**C′**) PLLA after laser activation and incubation in SBF.

Five types of PLLA-based samples were investigated in the research. The first was virgin PLLA film obtained in the casting extrusion process and investigated without any other modifications. The sample was used as a reference. The second and the third samples were PLLA subjected to UV (ArF, 193 nm) selective laser modification and incubated in air for either one day or seven days (denoted PLLA_UV_1d and PLLA_UV_7d, respectively). The fourth and the fifth were UV-laser modified PLLA immersed in SBF for one day and seven days (denoted PLLA_UV_SBF_1d and PLLA_UV_SBF_7d, respectively). The purpose of immersing a sample in SBF was: (1) to simulate conditions similar to those of the body and (2) obtain a ceramic layer on the laser irradiated surface as described earlier [32]. SEM pictures of the investigated samples are presented in Figure 1. No significant differences between PLLA, PLLA_UV_1d and PLLA_UV_7d can be seen (Figure 1A–C). All of the surfaces are very smooth. They have isolated inclusions left after the film preparation process. In conjunction with the results of optical profilometry (Figure 2), it is clearly visible that the laser modification process was successfully carried out below the material ablation threshold. However, slight cracks on the surface analyzed seven days after irradiation can be observed. Their occurrence is related to the relaxation of stresses formed in the polymer under the influence of laser irradiation. In contrast, PLLA_UV_SBF_1d and PLLA_UV_SBF_7d look significantly rougher (Figure 1D,E). Moreover, after seven days of incubation, the relative surface area of the polymer appears to be much greater. This is related to a much more in-depth hydrolysis process. To determine the exact surface values of the samples, additional experiments were performed using optical profilometry.

Optical profilometry measurements revealed that the average roughness (R_a_) of the unmodified reference PLLA surface was at the level of 25 nm (Table 1). The profile of the sample is shown in Figure 2A. After the UV-laser modification, the R_a_ parameter had actually decreased down to 21 nm for the sample left in air both for 1 day (Figure 2B) and for 7 days (Figure 2B′), which is considered to be almost the same roughness level (30 nm). The differences between unmodified and laser irradiated PLLA were not significant (within statistical error). The surface of SBF-immersed and irradiated PLLA was completely different (Figure 2C,C′). It was found that R_a_ (Table 1) has increased dramatically to almost 590 nm after incubation in SBF for 1 day, displaying a remarkably high roughness compared to the references. This effect is related to ceramic deposition on PLLA [32]. However, for the PLLA_UV_SBF_7d sample, R_a_ was lower (260 nm) than for PLLA_UV_SBF_1d. For the longer incubated sample (168 h), the surface coverage was fuller, while for short incubation (24 h), the coverage was observed only in a few places, which affected the resulting roughness. The area roughness (S_a_) trends were the same as for R_a_.

**Figure 3:**
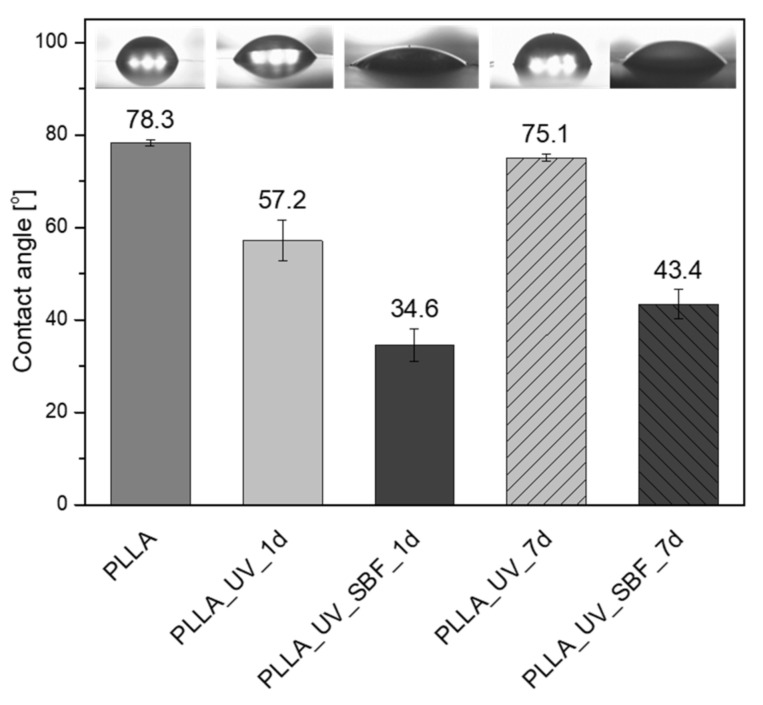
Contact angle measurements on modified surfaces.

Another important parameter is the water contact angle (Figure 3). In the case of this parameter, the hydrophobic surface having a water contact angle around 78° was measured for neat PLLA. The value is in good agreement with previous results [32]. For the irradiated sample PLLA_UV_1d the water contact angle decreased to 57°. The effect was observed earlier [32] and the values of water contact angles were comparable. After one week of incubation in air, the water contact angle for PLLA_UV_7d returned to almost the initial level of the reference PLLA (75°), which suggests that the modification effect was not stable and changed over time. This effect can also be related to adsorption of contaminants by the UV-activated PLLA surface. The water contact angle of the irradiated and SBF-incubated sample PLLA_UV_SBF_1d was around 35°, which is related to the surface topography (high roughness of the sample surface) as well as mineral depositions. A similar effect was observed earlier, despite the different conditions of laser irradiation [32]. Surprisingly, for the PLLA_UV_SBF_7d, the water contact angle rose to 43°. This effect might be correlated with the difference in surface roughness. However, the difference between PLLA_UV_SBF_1d and PLLA_UV_SBF_7d was only slightly greater than the standard deviation.

### 2.2. X-ray Photoelectron Spectroscopy Analysis

**Figure 4:**
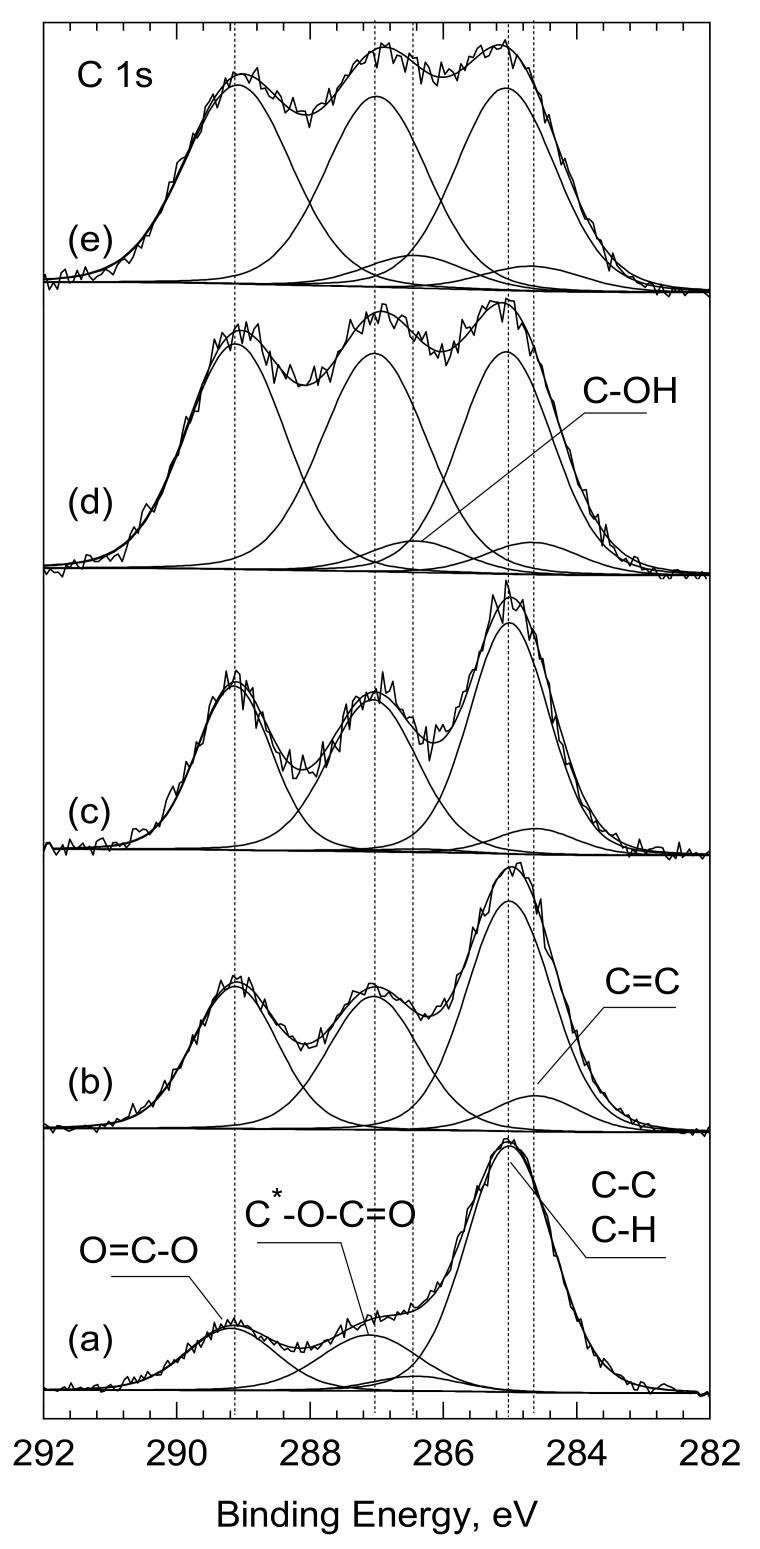
C1s core level spectra for the surfaces of: (**a**) PLLA, (**b**) PLLA_UV_1d, (**c**) PLLA_UV_7d, (**d**) PLLA_UV_SBF_1d, (**e**) PLLA_UV_SBF_7d analyzed as ‘received’. *—the asterisk indicates which carbon atom the peak applies to.

In line with the chemical structure of PLLA, three components were distinguished within the carbon spectrum C1s. The peak at 285.0 eV characteristic for the C-C bond in the chemical structure of PLLA was used as a reference. The other two components, at 287 eV and 289.1 eV, were assigned to the C-O-C=O and O=C-O bonds, respectively. Their ratio of 1:1 was consistent with the chemical structure of PLLA. At the same time, the share of the C-C/C-H bond on the surface of PLLA analyzed as ‘received’, was significantly higher and amounted to 63.5% (versus 33.3% for bulk) (Table 2, Figure 4). This excess amount of the C-C/C-H bonds is typically due to adventitious/contamination carbon. Moreover, for best fitting, an additional peak at 286.4 eV was inserted into the C 1s spectrum. Its share was estimated at 3.9% and was assigned to the OH groups adsorbed on the surface of PLLA.

After laser treatment (PLLA_UV_1d), the shape of the C 1s spectrum changed significantly. First, the number of the C-C/C-H bonds was reduced from 63.5 to 41.9% and there were almost no C-OH groups (Figure 4b and Table 2). While the energy of the laser beam was high enough to desorb the OH groups from the surface, it was certainly too low to clean the PLLA surface of the absorber carbon contamination. Therefore, it is natural to conclude that following the UV-laser irradiation of PLLA, the polymer was partially degraded. If we assume that the photodegradation of PLLA follows the Norrish II mechanism [33,34,35,36], then both the quantitative analysis and the deconvolution of C 1s spectra from the PLLA_UV_1d, as well as PLLA_UV_7d, are consistent with this. According to Norrish, after breaking the -O-C- chain, two lower molecular weights with new C=C and O-H bonds appear [34]. The share of the former, produced at the expense of the C—C bonding, was estimated at 6.6% in relation to total carbon. On the other hand, the newly formed—O-H group forms the end of the previously existing O=C-O-(C_ part, in which the binding energy C 1s (289.1 eV) and remained unchanged. The bond ratio of (O-C-O): (C-O) = 1:1 was maintained. It should also be noted that 6.6% of the new C=C bond does not compensate for the greater drop in the share of the C-C/C-H bond: from 63.5 to 41.9%. However, as mentioned earlier, this amount also includes the contaminating carbon, which decreased after breaking the PLLA molecules and after the interior PLLA (bulk) was exposed.

Moreover, for both samples immersed in SBF (PLLA_UV_SBF_7d and PLLA_UV_SBF_7d), additional Ca 2p measurements were conducted. A small amount of calcium was found on the surface of irradiated PLLA (Table 2 and Figure S6), which confirmed the deposition of calcium on the immersed samples.

### 2.3. Cellular Response

**Figure 5:**
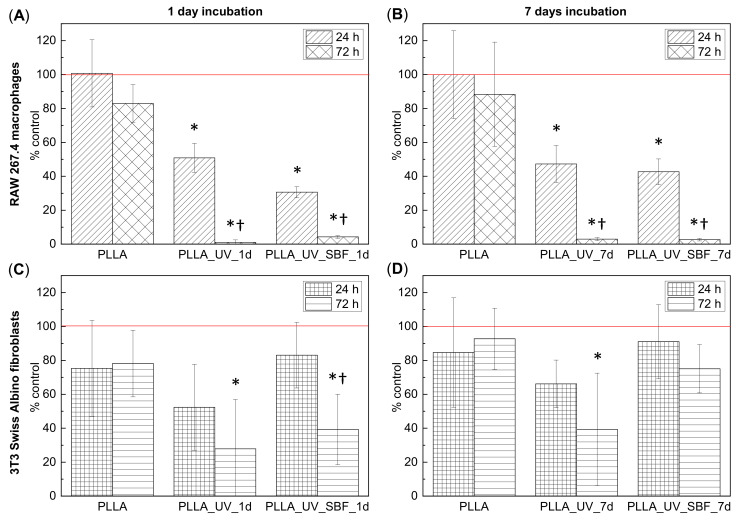
(**A**,**B**) Viability of the RAW 267.4 macrophages cultured for 24 h and 72 h on samples incubated for 1 day (**A**) and samples incubated for 7 days (**B**): unmodified polylactide (PLLA), laser-treated polylactide (PLLA_UV), and SBF- and laser-treated polylactide (PLA_UV_SBF) compared to the control (polystyrene). (**C**,**D**) Viability of the 3T3 Swiss Albino fibroblasts cultured for 24 h and 72 h on samples incubated for 1 day (**C**) and samples incubated for 7 days (**D**): unmodified polylactide (PLLA), laser-treated polylactide (PLA_UV), and SBF- and laser-treated polylactide (PLA_UV_SBF) compared to the control (polystyrene). The red line corresponds to 100% of cells cultured on the control polystyrene. * indicates a significant difference compared to PLLA for the same duration of exposure. † indicates a significant difference between different times of exposure (24 vs. 72 h). *p* < 0.05 was considered significant.

Figure 5A,B show the results of the cytotoxicity assessment in RAW 267.4 cells cultured on different PLLA surfaces. Unmodified PLLA was very well tolerated by cells during exposure for 24 h; however, longer incubation (72 h) seemed to slightly impede cell proliferation as compared to polystyrene control. On the other hand, the surfaces of PLLA_UV_1d and PLLA_UV_SBF_1d induced distinct, time-dependent cytotoxicity. After 72 h of incubation only a few living cells were left. Longer incubation in SBF and additional washing steps (PLLA_UV_SBF_7d) did increase the roughness and thickness of the mineral layer but decreased the cytotoxic response of the macrophages only slightly, and only in the case of short incubation time (24 h). Ageing had no effect at all on the cytotoxicity for the irradiated polylactide. Figure 5C,D summarizes the results of cytotoxicity assessment in the 3T3 Swiss Albino fibroblasts. On unmodified PLLA, these cells seemed to proliferate less efficiently compared to macrophages, irrespective of the incubation period. However, PLLA_UV_1d and PLLA_UV_SBF_1d seemed to be much better tolerated by these cells. Although variability in the fibroblast response to substrates was rather high, the tendency in both cell lines was clear in that the activation of the PLLA surface by laser irradiation led to a distinct increase in cytotoxicity, and the treatment with SBF has some preventive effect on this harmful impact. It should be mentioned that the fresh laser-treated material had a distinct smell, suggesting the presence of volatile degradation products. It seems quite likely that these compounds contributed to the material toxicity. For the irradiated polylactide PLLA_UV_7d, ageing decreased cytotoxicity only slightly; however, aged PLLA_UV_SBF_7d was visibly better tolerated by fibroblasts even in the case of longer incubation (72 h). The cell-dependent differences in response to the SBF-treated polylactide suggest the involvement of different mechanisms of toxicity. A thicker ceramic layer and washing away of some laser-induced degradation products seem to provide a sufficiently biocompatible environment for fibroblasts. The response of macrophages seemed different. It is known that cell-mediated degradation of polylactide leads to local accumulation of lactic acid and acidosis [37]. Since macrophages are more efficient in degrading PLLA compared to fibroblasts, they may also be more exposed to the cytotoxic effects of local acidosis. The UV treatment may actually predegrade the surface of the polymer [32,38] making the cell-mediated degradation process faster and the accumulation of cytotoxic products higher. Neither an additional washing nor a thicker ceramic layer seemed to suffice to prevent this effect. Low pH on the macrophage-polymer interface may easily lead to dissolution of the deposited ceramic layer limiting its protective potential still seen in fibroblasts.

**Figure 6:**
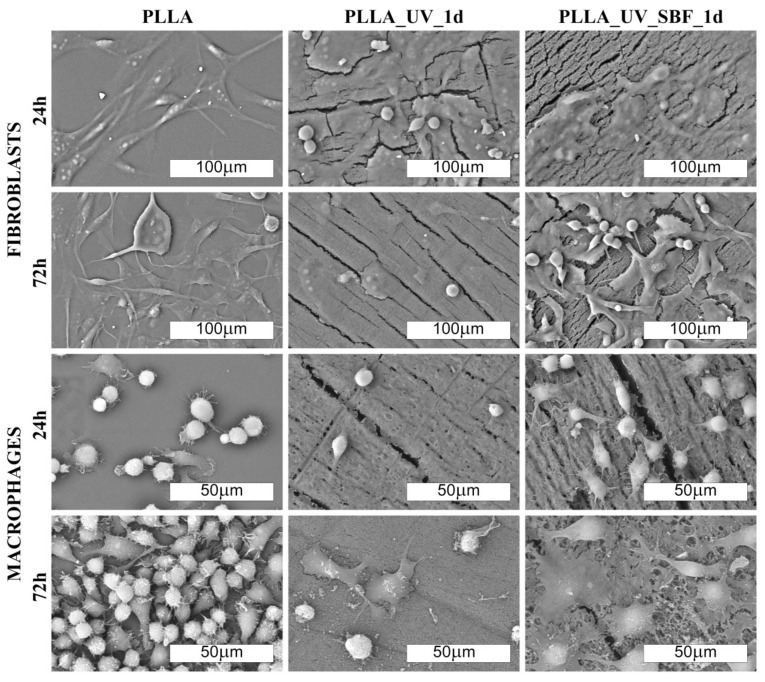
SEM images of fibroblast and macrophage’ colonies (500× and 1000× magn. respectively) on the laser-modified samples after one day of incubation in air (PLLA_UV_1d) and in SBF (PLLA_UV_SBF_1d) as compared to the unmodified PLLA (PLLA).

**Figure 7:**
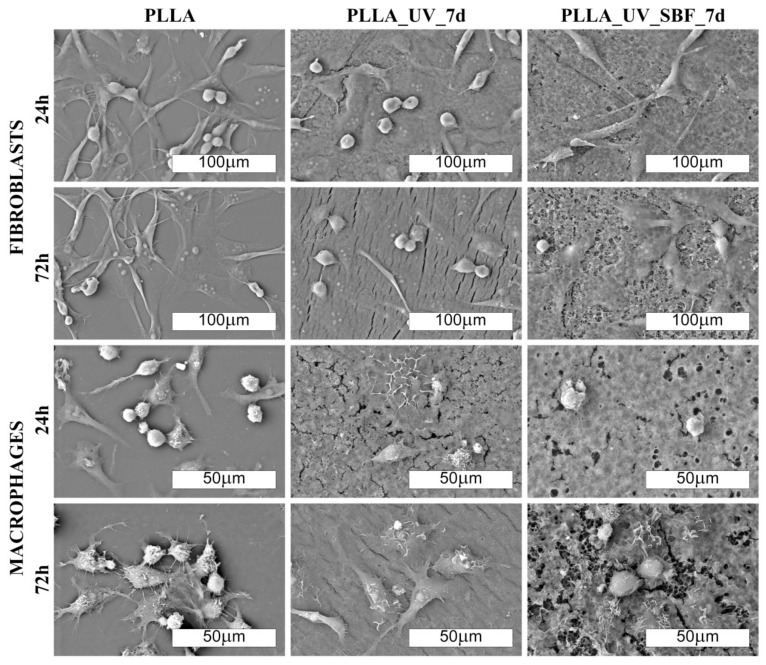
SEM images of fibroblast and macrophage colonies (500× and 1000× magn., respectively) on the laser-modified samples after one day of incubation in air (PLLA_UV_7d) and in SBF (PLLA_UV_SBF_7d) compared to the unmodified PLLA (PLLA).

The morphology of cells cultured on PLLA and modified materials is shown in Figure 6 and Figure 7 (SEM). There was no distinct impact of the substrate on the morphology of cells; however, macrophages seemed to be more rounded on the PLLA surface. On all of the modified samples, macrophages seemed to be flatter, which suggests a slightly higher surface of adherence compared to PLLA (especially for 72 h). It is known that macrophages are the primary cells involved in the degradation of polylactide implanted under in vivo conditions [37]; therefore, this slight change in the phenotype may reflect participation in the attempted degradation of the surface of PLLA.

Morphological differences were seen in the 3T3 Swiss Albino fibroblasts (Figure 6 and Figure 7). For PLLA, well spread cells attached to the surface and evenly distributed were observed (both for 24 and 72 h). For the PLLA_UV_1d and PLLA_UV_7d, the 3T3 morphology was rather different for that on PLLA. Some cells were flat and attached to the surface, but others were round-shaped with no filopodia spread. This effect was similar to the one described by Slepička [39] for laser irradiated polyhydroxybutyrate. Finally, the morphology of 3T3 on PLLA_UV_SBF_1d and PLLA_UV_SBF_7d (both 24 and 72 h) resembled that in the reference PLLA. In this case, cells were mostly elongated along the surface, displaying good adhesion to the surface. Nevertheless, a few round cells could also be observed. For the samples that spent 7 days in SBF (PLLA_UV_SBF_7d and PLLA_UV_SBF_7d), the 3T3 morphology was similar to that of neat PLLA.

Surface wettability is yet another factor that may have a significant impact on the cellular response of macrophages and fibroblasts on the analyzed samples. William G. Brodbeck et. al. showed that cells from the macrophage lineage showed a greater affinity to hydrophobic surfaces because an increase in hydrophilicity promoted apoptosis processes [40]. Hence, the conclusion that the modifications carried out in the experiment may be unfavorable for macrophage cells due to the excessive increase in surface wettability. The situation is different with fibroblasts. It has been shown several times that an increase in surface hydrophilicity is beneficial, and it is also worth mentioning that all the UV-modified samples exhibited cracks (Figure 6 and Figure 7).

Although these hypotheses may explain the differences seen in both cell models, they need to be supported by further experiments, as they may lead to important conclusions in terms of biomaterial design.

The selective effect on macrophage and fibroblast viability may have a potentially beneficial implication for the design of implant coverings based on the technique described in this study. Since fibroblasts are responsible for the healing process and the formation of scar tissue around the surgically inserted implant, their dominance over macrophages may contribute to the lower inflammatory response mediated by these cells. However, this hypothesis requires further studies performed using in vivo models where the complex processes of healing and inflammation are reflected in full spectrum.

Comment: Fibroblast cells adhere well to the unmodified PLLA surface. On laser-modified samples, some of the cells become round-shaped, which indicates a lower adhesion to the surface due to unfavorable conditions. The situation changes in favor on the samples after modification in SBF. Macrophages, in turn, do not show significant differences in morphology on the modified surfaces. However, their density is evidently lower on them.

Comment: Fibroblast cells adhered well to the unmodified PLLA surface. On laser-modified samples, some of the cells became round-shaped, which indicates a lower adhesion to the surface due to unfavorable conditions. The situation changed in favor of the samples after modification in SBF. Macrophages, in turn, did not show significant differences in morphology on the modified surfaces. However, their density was evidently lower.

## 3. Materials and Methods

### 3.1. Materials and Preparation of Samples

In this study we used poly(L-lactide), Resomer L210S (M_n_ = 304,020 g/mol, PDI = 1.96, 100% L-lactide unit fraction of PLLA – own measurements), obtained from Evonik (Germany). PLLA films were prepared according to the methodology described in our previous articles [32,41]. Briefly, PLLA granulates were extruded using a Labtech Engineering (Sweden/Thailand) conical, single screw micro-extruder equipped with a flat die (300 μm high and 75 mm wide). The screw diameter was in the range of 18–8 mm (from hoper to die). The extrusion temperature was set at 200 °C and the screw speed was 200 rpm. After extrusion, the film was cooled down in air to 50 °C, transferred to the collecting system and wound on a spool. The obtained films were about 150 micrometers. The molecular weight determined for the polymer film was M_n_ = 145,600 g/mol and PDI = 2.3.

Also SBF was prepared in the same way as described earlier [32]. The method was initially taken from the research of Kokubo [25].

### 3.2. Laser Modification—PLLA Surface Activation

**Figure 8:**
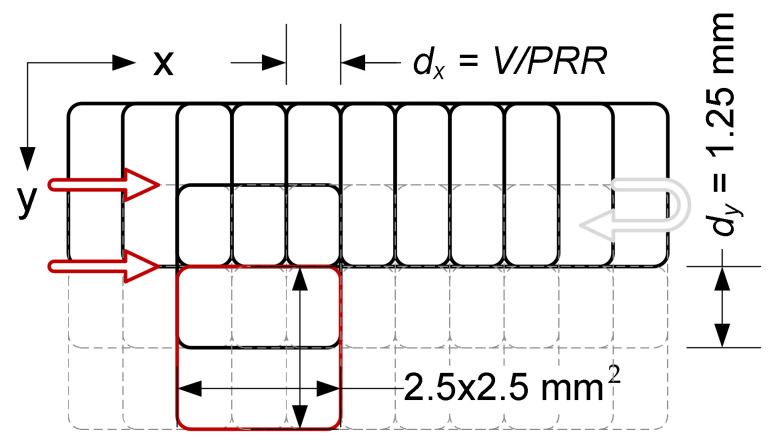
Scheme of unidirectional exposure of PLLA samples (showing the way the pulses overlaps in both directions) with the ArF excimer laser.

The samples were irradiated using a ProMaster laser micromachining system by Optec^®^ (Optec, Frameries, Belgium) equipped with an ArF excimer laser (model SP300-SI-196 by ATL Lasertechnik, Wermelskirchen, Germany) with a wavelength of 193 nm, pulse duration 5–6 ms, pulse repetition rate up to 300 Hz and the energy of a single pulse up to 8 mJ. In order to eliminate the effect of residual heat accumulation from individual pulses, the pulse repetition rate in the experiment was limited to 100 Hz. The samples were irradiated without an imaging lens using a single pulse energy of 6 mJ (which guaranteed the stability of laser operation) and a scan speed of 0.5 mm/s. To reduce the photolytic degradation of the surface, an external optical attenuator (T ≈ 5%) was used, limiting the energy of a single pulse to 273 µJ (measured in the working field, taking into account the total losses in the beam path). The beam size on the surface of sample was about 2.5 × 2.5 mm^2^ FWHM (4.0 × 4.0 mm^2^ D4σ). The applied single pulse laser fluence was estimated at 4.4 mJ/cm^2^. This value was over an order of magnitude lower than the ablation threshold, and at the same time allowed us to induce selective mineralization of the polymer. Single pulse fluence is defined as the pulse energy divided by the spot surface of the laser beam. The samples were irradiated in a unidirectional raster mode (line by line) with hatching equal to half the height of the laser spot (Figure 8) The high overlapping of the defocused laser beam (1000 pulses at every point of the surface) ensured that the irradiation of the sample was homogeneous.

### 3.3. Incubation of PLLA in SBF

Samples of irradiated PLLA were immersed in SBF and incubated in an air dryer for 1 day (24 h) and 7 days (168 h) at 37 °C. When incubating the irradiated samples in SBF, the proportion of 16 mL SBF per 1 cm^2^ of the irradiated area was maintained. During long-term incubation (168 h), simulated body fluid was replaced with a fresh SBF after 48 and 96 h. The irradiated and SBF-incubated samples became selectively cloudy only in the irradiated area of the PLLA surface. The nonirradiated PLLA showed no optical changes even after 168 h in SBF. After incubation, the samples were washed several times in deionized water and dried overnight at 40 °C. Neat PLLA was used as a reference as well as UV-irradiated PLLA incubated in air for 24 and 168 h. Sample descriptions are summarized in Table 3.

### 3.4. X-ray Photoelectron Microscopy (XPS)

X-ray photoelectron spectroscopy measurements were carried out using a SPECS PHOIBOS 100 spectrometer (SPECS, Berlin, Germany) with an Mg anode. The base pressure in the UHV chamber was better than 5 × 10^−10^ mbar. The recorded spectra were processed and fitted using SPECLAB 2 and CasaXPS software. The Gaussian-Lorentzian curve profile and Schirley baseline were applied. The C1s peak at 285.0 eV for C-C/C-H bonds was used as a reference for all spectra. All the deconvolutions are shown in Figures S1–S5.

### 3.5. Scanning Electron Microscopy (SEM) 

Scanning electron microscopy was used for cell observations on samples as well as for the surface morphology of modified films. For this purpose, a Zeiss Sigma 500 VP Scanning Electron Microscope (Zeiss, Oberkochen, Germany) in the BSE (Backscattered-Electron Imaging) detector mode was used. The microscope operated at 20 kV in a vacuum below 10^−5^ mbar, and the magnification ranged from 100× to 2000×. Samples were covered with Au (sputter current: 40 mA, sputter time: 50 s) using a QUORUM machine and dried before measurements at the critical point in a Leica EM CPD300 dryer.

The SEM analysis was performed to visualize the cell colonies. i.e., macrophages (RAW 264.7) and fibroblasts (3T3 Swiss Albino) on the modified surfaces. Samples with macrophages and fibroblasts were fixed using a 3% glutarate (POCH, Gliwice, Poland) for 15 min at room temperature. Next, the samples were rinsed twice with phosphate buffer (Sigma-Aldrich, Saint Louis, Missouri, USA) for the purpose of fixative elimination. Lastly, dehydration in increasing concentrations of ethanol (25, 60, 95, 100%) for 5 min in each solution was carried out. After that, the samples were further prepared according to the procedure for surfaces without biological material (described above).

### 3.6. Profilometry

In order to accurately analyze the surface topography of the tested samples, a confocal Olympus Lext 5000 microscope equipped with a diode laser generating 405 nm was used. A 100× lens was used for observation.

### 3.7. Water Contact Angle Measurement

The measurements were conducted with a PGX goniometer in static mode. At least 10 measurements were performed for each sample, and the mean value, as well as the standard deviation, were calculated for these results. Deionized water was used.

### 3.8. Cell Lines and Culture

Our cytotoxicity assessment was carried out on murine macrophage (RAW 264.7) and fibroblast (3T3 Swiss Albino) cell lines (Manassass, Virginia, USA). Although immortalized by viral vectors, these cells retain many physiological features and are considered to be proper models of normal cells [42,43]. The choice of these two models was dictated by the possible biomedical application of the biomaterials under study as the implant covering surface. Under in vivo conditions, fibroblasts are a major component of the granulation tissue that eventually contributes to the encapsulation of the implant inserted in a surgical procedure. On the other hand, macrophages are the primary cells involved in the immunological reaction of the body against implants [37,44]. Therefore, it is important to assess the response of these two types of cells to a new candidate biomaterial as early in the development process as possible.

Both cell lines were cultured in a high glucose DMEM medium (prepared in the Institute of Immunology and Experimental Therapy, Wrocław, Poland) supplemented with 10% fetal bovine serum (FBS, Gibco, USA), L-glutamine (Sigma-Aldrich, Gillingham UK), sodium pyruvate (Sigma-Aldrich, Taufkirchen, Germany) and antibiotics (penicillin and streptomycin, Sigma-Aldrich, Taufkirchen, Germany). Cytotoxicity, expressed as the effect on cell growth on the investigated surfaces, was measured by the trypan blue exclusion assay (TBEA) based on counting the actual number of living cells [45]. The MTT assay, which is significantly less labour-intensive, was found to be inappropriate due to the spontaneous oxidation of the dye to formazan by some of the surfaces.

### 3.9. Trypan Blue Exclusion Assay

Before the in vitro assay, laser-cut discs made of the PLLA materials were placed in 24-well-plates (TPP, Switzerland) and tightened with tailor-made sterile Teflon O-rings that fitted precisely to the wells and prevented migration of cells under the material. Next, all wells were preincubated with fresh culture medium for 30 min at 37 °C. After that, RAW 264.7 cells were seeded at a density of 50 × 10^3^ (for the 24 h incubation) or 20 × 10^3^ (for the 72 h incubation) per well. The seeding density of the 3T3 Swiss Albino fibroblasts was 40 × 10^3^ (for the 24 h incubation) and 20 × 10^3^ (for the 72 h incubation) per well. In all cases, cells were incubated at 37 °C in a humidified atmosphere of 5% CO_2_. After the incubation was completed, the culture medium was removed and cells were washed with phosphate buffered saline to remove dead and unattached cells. Next, the remaining cells were detached using 0.25% trypsin in EDTA (Sigma-Aldrich, Saint Louis, MO, USA ) mixed with a 0.4% trypan blue solution (Sigma-Aldrich) and counted in a haemocytometer. In this assay, cells that do not stain blue are considered viable. Cell viability was calculated as a percent of the control. Each assay was made in triplicate, and the results are presented as a mean value (±SD) of three independent assays. Within a single replicate, each material was tested in duplicate and cells in each well were calculated twice. Therefore, for a single type of surface a series of 12 values was used to arrive at the final results.

The statistical significance of the differences was determined by the Student’s t-test with significance threshold set at *p* < 0.05 (Microsoft Excel, Redmond, WA, USA).

## 4. Conclusions

In this study we prepared PLLA extruded films and subjected them to modifications using an UV-excimer laser. The samples were subsequently incubated either in air (for 1 day and for 7 days) or in SBF (for 1 day and 7 days). All the samples were compared to the unmodified PLLA. It was found that the surface of PLLA after UV treatment and incubation in SBF displayed an over ten times higher roughness (Ra) compared to the unmodified PLLA and the UV-irradiated PLLA. The higher roughness values were related to the deposition of ceramics on the surface. The PLLA_UV_SBF_1d and PLLA_UV_SBF_7d samples revealed reduction in the water contact angle compared to neat PLLA showing higher hydrophilicity. All of the modified PLLA surfaces proved cytotoxic for macrophages irrespective of the deposition of ceramic layer and the duration of incubation in SBF. For fibroblasts, however, improved biocompatibility was seen with the ceramic layer present compared to the surface treated only with UV. Moreover, with longer incubation in SBF and a thicker ceramic deposit, biocompatibility was restored up to the level of the control PLLA.

As indicated in previous research, UV-laser modification accelerates the degradation of PLLA. In the context of its applications in tissue engineering, such modification adversely affects the material. This research shows that this negative effect can be partially compensated by the use of bioceramisation.

## Figures and Tables

**Table 1:** Roughness parameters of analyzed samples based on the surface profiles (S_a_, S_z_) and the single line profiles (R_a_, R_z_) shown in Figure 2.

Sample	S_a_ [nm]	S_z_ [μm]	R_a_ [nm]	R_z_ [μm]
PLLA	49_±3_	0.80_±0.06_	25_±3_	0.16_±0.04_
PLLA_UV_1d	36_±7_	0.45_±0.07_	21_±3_	0.15_±0.02_
PLLA_UV_SBF_1d	515_±55_	5.52_±0.58_	588_±62_	3.62_±0.45_
PLLA_UV_7d	38_±4_	0.83_±0.15_	30_±3_	0.15_±0.03_
PLLA_UV_SBF_7d	416_±29_	9.72_±1.09_	258_±55_	1.80_±0.33_

X_a_—Average roughness, X_z_—Arithmetical mean height, the presented values are the average of five measurements.

**Table 2:** Fraction of carbon functional groups from the high-resolution C 1s XPS peak on the surface of pristine PLLA, after laser irradiation and treatment in SBF.

	C-C/C-H 285.0 eV	C-O-C=O 287.0 eV	O=C-O 289.1 eV	C-OH 286.4 eV	C=C 284.6 eV	O/C	Ca/C
Theoretical ratio	33.3	33.3	33.3	-	-	0.67	-
PLLA	63.5	16.3	16.3	3.9	0	0.25	-
PLLA_UV_1d	41.9	25.8	25.6	0.1	6.6	0.43	-
PLLA_UV_7d	39.1	29.0	26.2	0.6	5.1	0.48	
PLLA_UV_SBF_1d	28.9	31.8	31.0	4.1	4.2	0.53	0.005
PLLA_UV_SBF_7d	30.3	29.3	31.8	4.9	3.7	0.50	0.005

**Table 3:** Samples description.

Samples Description	Laser Treatment	Incubation in Air	Incubation in SBF
PLLA	−	-	-
PLLA_UV_1d	+	1 day (24 h)	-
PLLA_UV_7d	+	7 days (168 h)	-
PLLA_UV_SBF_1d	+	-	1 day (24 h)
PLLA_UV_SBF_7d	+	-	7 days (168 h)

## Data Availability

The data presented in this study are available within the article. Other data that support the findings of this study are available upon request from the corresponding authors.

## Supplementary Materials

The following are available online at https://www.mdpi.com/article/10.3390/ijms22168436/s1.
