# Supplementary material for: Cytotoxicity Study of UV-Laser-Irradiated PLLA Surfaces Subjected to Bio-Ceramisation: A New Way towards Implant Surface Modification
Source: Int J Mol Sci. 2021 Aug 5;22(16):8436. doi: 10.3390/ijms22168436 (PMC8395104; doi:10.3390/ijms22168436)
Supplement: Supplementary file 1 [file ijms-22-08436-s001.zip › ijms-1285774-supplementary.pdf]

*Article*

# Cytotoxicity Study of UV-Laser-Irradiated PLLA Surfaces Subjected to Bio-Ceramisation: A New Way towards Implant Surface Modification

**Konrad Szustakiewicz [1,*], Bartłomiej Kryszak [1,*], Paulina Dzienny [2], Błażej Poźniak [3], Marta Tikhomirov [3], Viktoria Hoppe [4], Patrycja Szymczyk-Ziółkowska [4], Włodzimierz Tylus [5], Michał Grzymajło [1], Agnieszka Gadomska-Gajadhur [6] and Arkadiusz J. Antończak [2]**

[1] Department of Polymer Engineering and Technology, Faculty of Chemistry, Wrocław University of Science and Technology (WUST), Wyb. Wyspiańskiego 27, 50-370 Wrocław, Poland; michal.grzymajlo@pwr.edu.pl
[2] Laser and Fiber Electronics Group, Faculty of Electrical Engineering, Wrocław University of Science and Technology, 50-370, Wrocław, Poland;
paulina.dzienny@pwr.edu.pl (P.D.); arkadiusz.antonczak@pwr.edu.pl (A.A.)
[3] Department of Pharmacology and Toxicology, Faculty of Veterinary Medicine, Wrocław University of Environmental and Life Sciences, ul. Norwida 25, 50-375 Wrocław, Poland;
blazej.pozniak@upwr.edu.pl (B.P.); marta.tikhomirov@upwr.edu.pl (M.T.)
[4] Centre for Advanced Manufacturing Technologies, Faculty of Mechanical Engineering, Wrocław University of Science and Technology (WUST), Łukasiewicza 5, 50-370 Wrocław, Poland;
viktoria.hoppe@pwr.edu.pl (V.H.); patrycja.e.szymczyk@pwr.edu.pl (P.S.-Z.)
[5] Department of Advanced Material Technologies, Faculty of Chemistry, Wrocław University of Science and Technology, 50-370, Wrocław, Poland;
wlodzimierz.tylus@pwr.edu.pl
[6] Faculty of Chemistry, Warsaw University of Technology, ul. Noakowskiego 3, 00-664 Warsaw, Poland;
agadomska@ch.pw.edu.pl
* Correspondence: konrad.szustakiewicz@pwr.edu.pl (K.S.); bartlomiej.kryszak@pwr.edu.pl (B.K.)

## Supporting Info

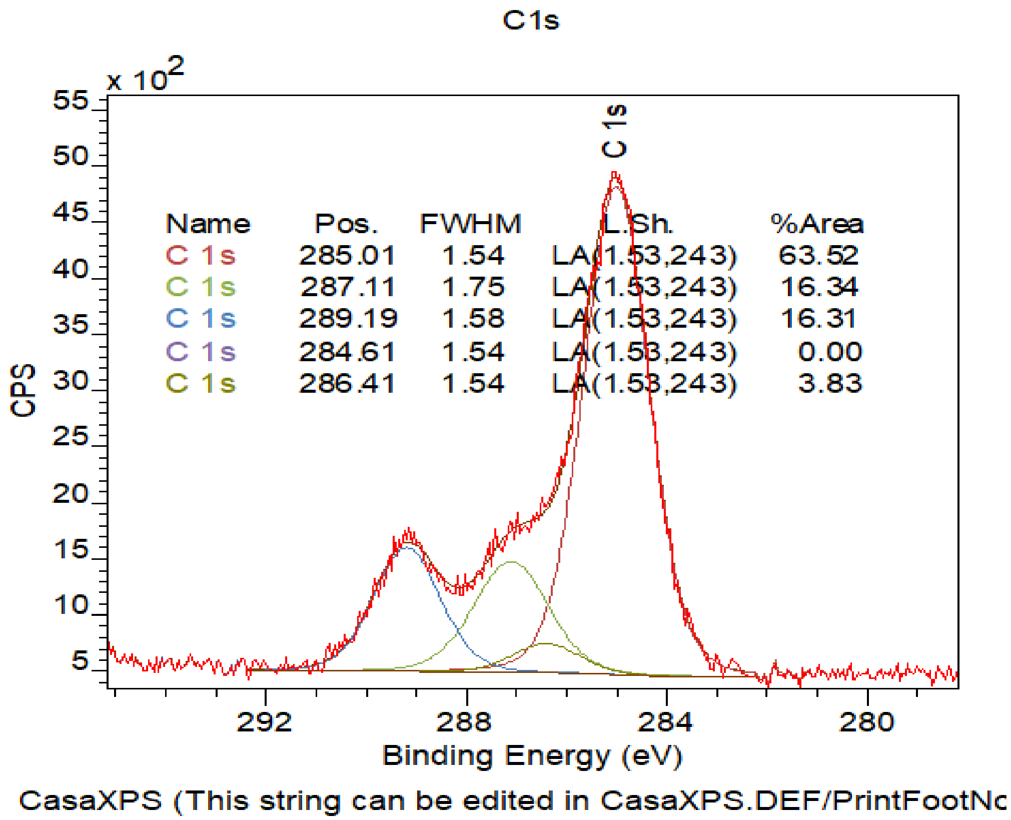


Figure S1. XPS C1s spectra of PLLA foil

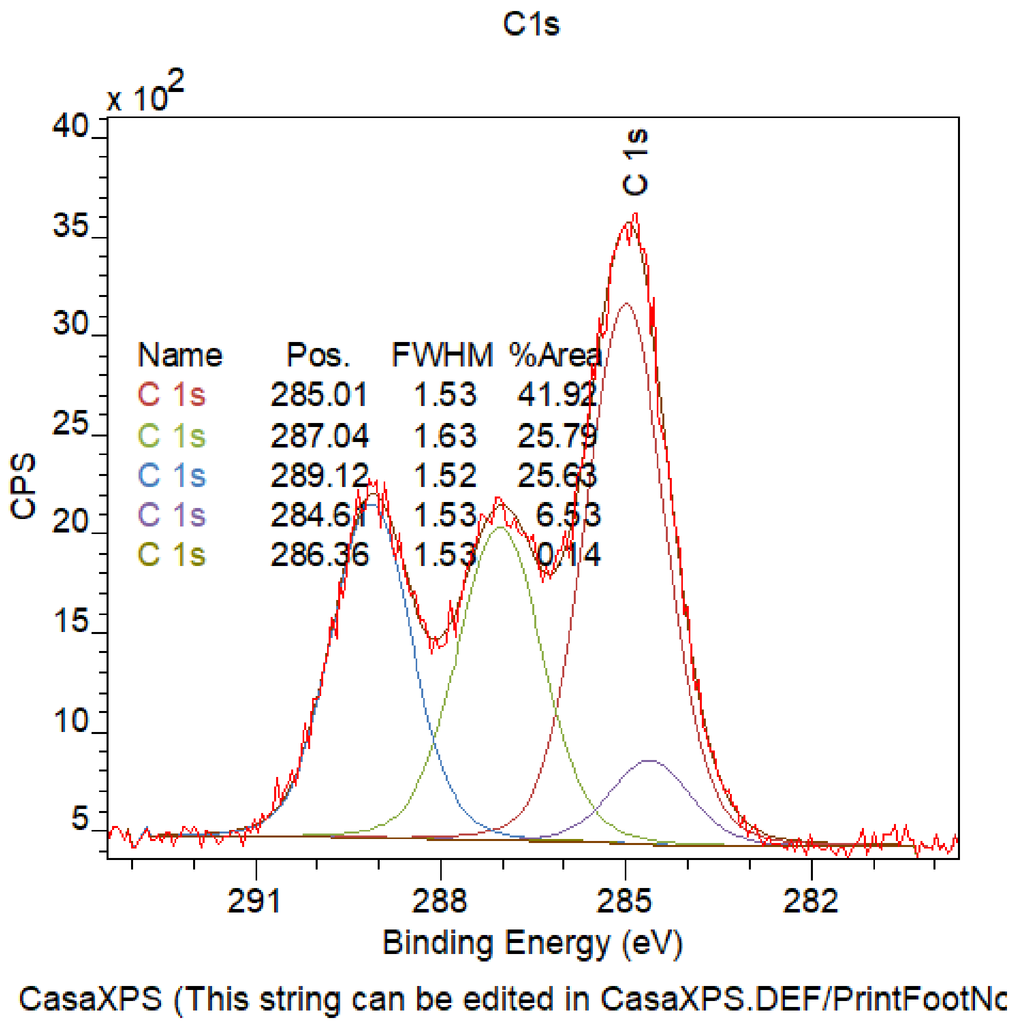


Figure S2. XPS C1s spectra of PLLA_UV_1d

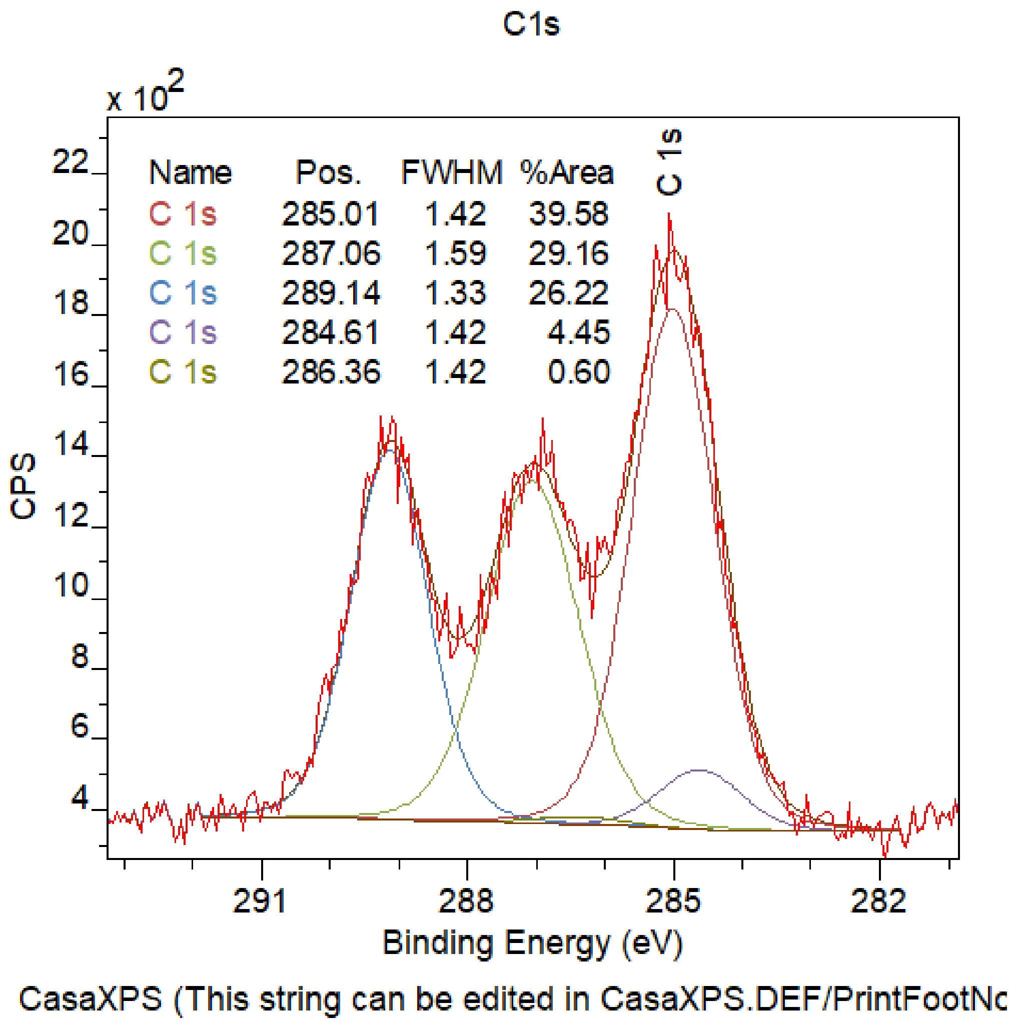


Figure S3. XPS C1s spectra of PLLA_UV_7d

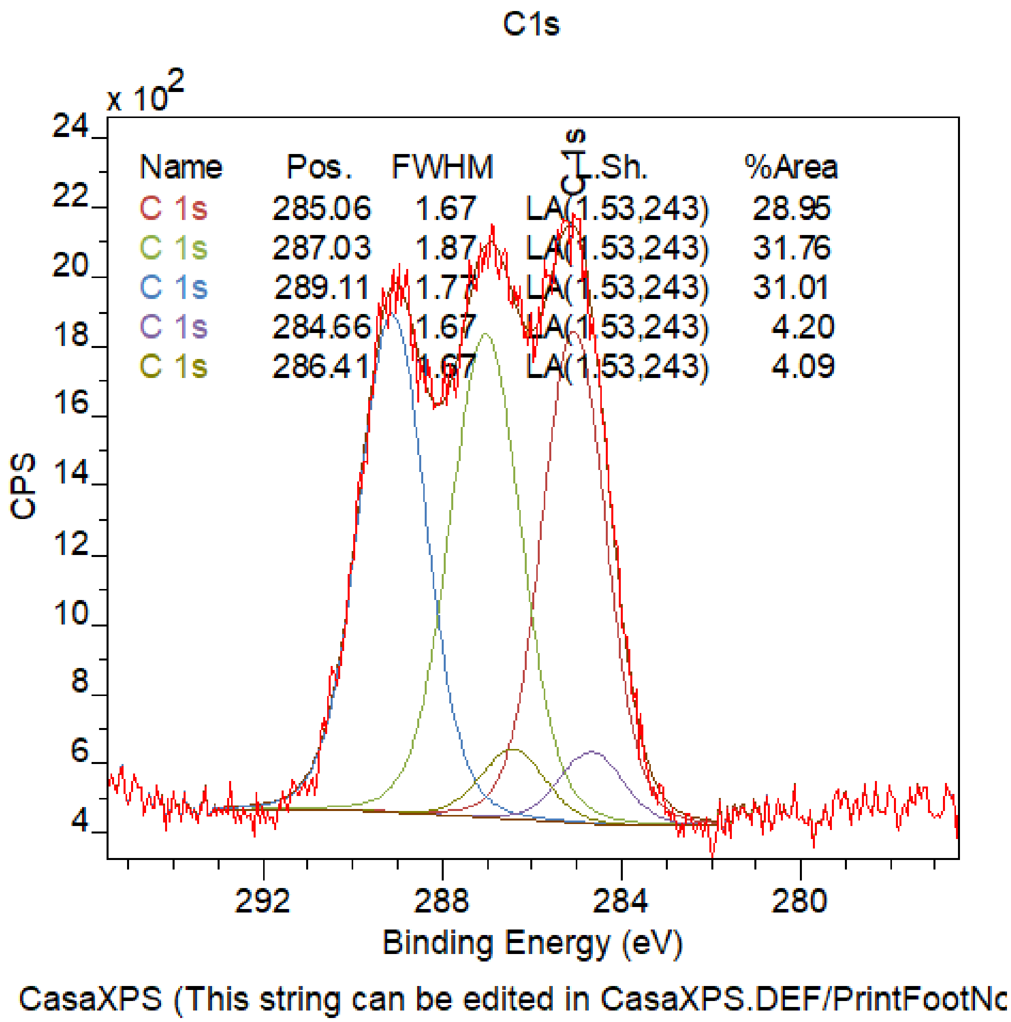


Figure S4. XPS C1s spectra of PLLA_UV_SBF_1d

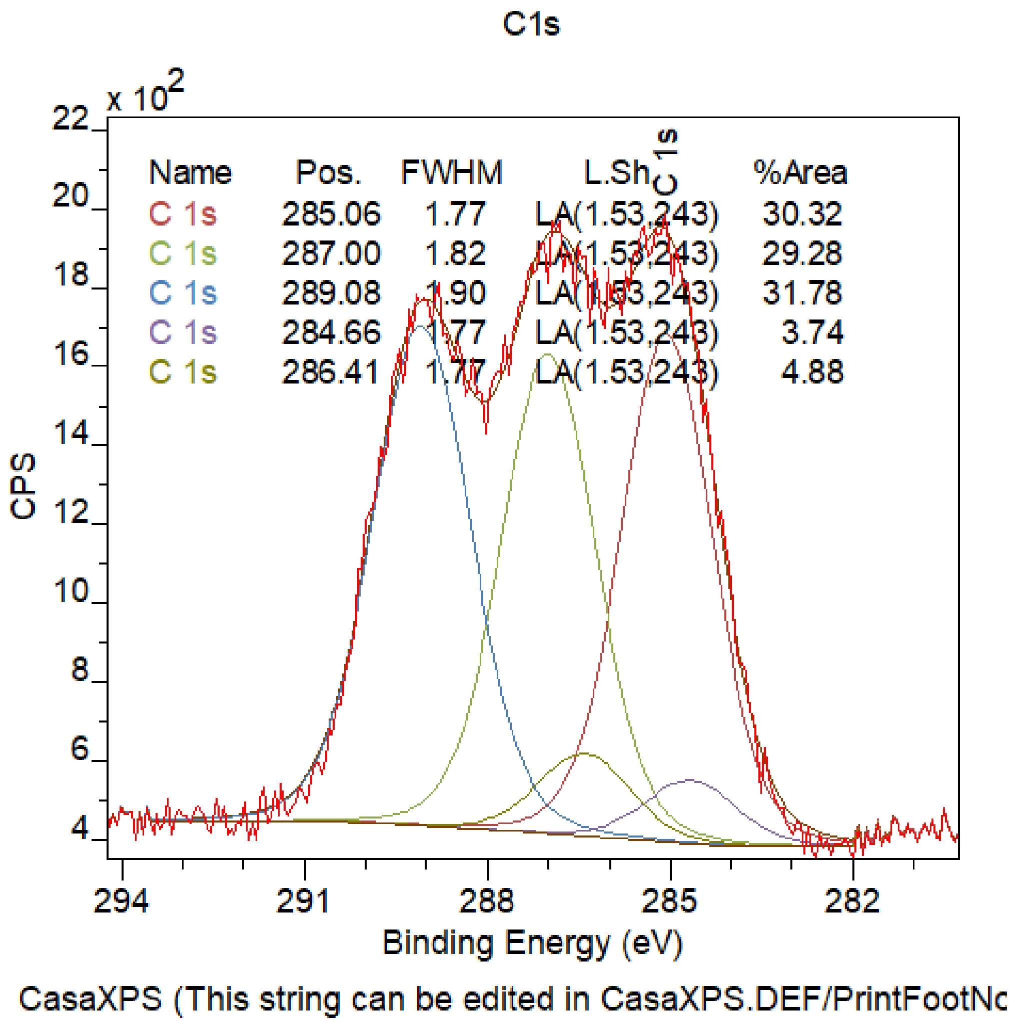


Figure S5. XPS C1s spectra of PLLA_UV_SBF_7d

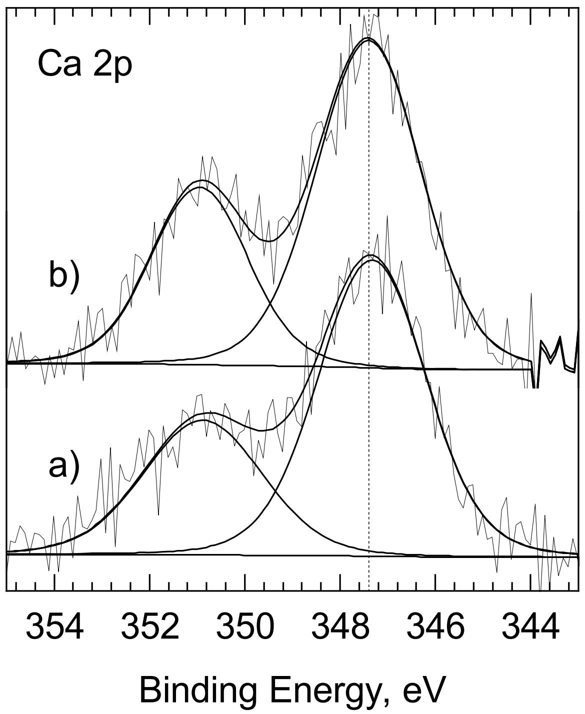

Figure S6. Ca 2p core level spectra for a) PLLA_UV_SBF_1d and b) PLLA_UV_SBF_7d
